# Supplementary figures and images for: Emergent Temporal Signaling in Human Trabecular Meshwork Cells: Role of TRPV4-TRPM4 Interactions
Source: Front Immunol. 2022 Mar 31;13:805076. doi: 10.3389/fimmu.2022.805076 (PMC9008486; doi:10.3389/fimmu.2022.805076)

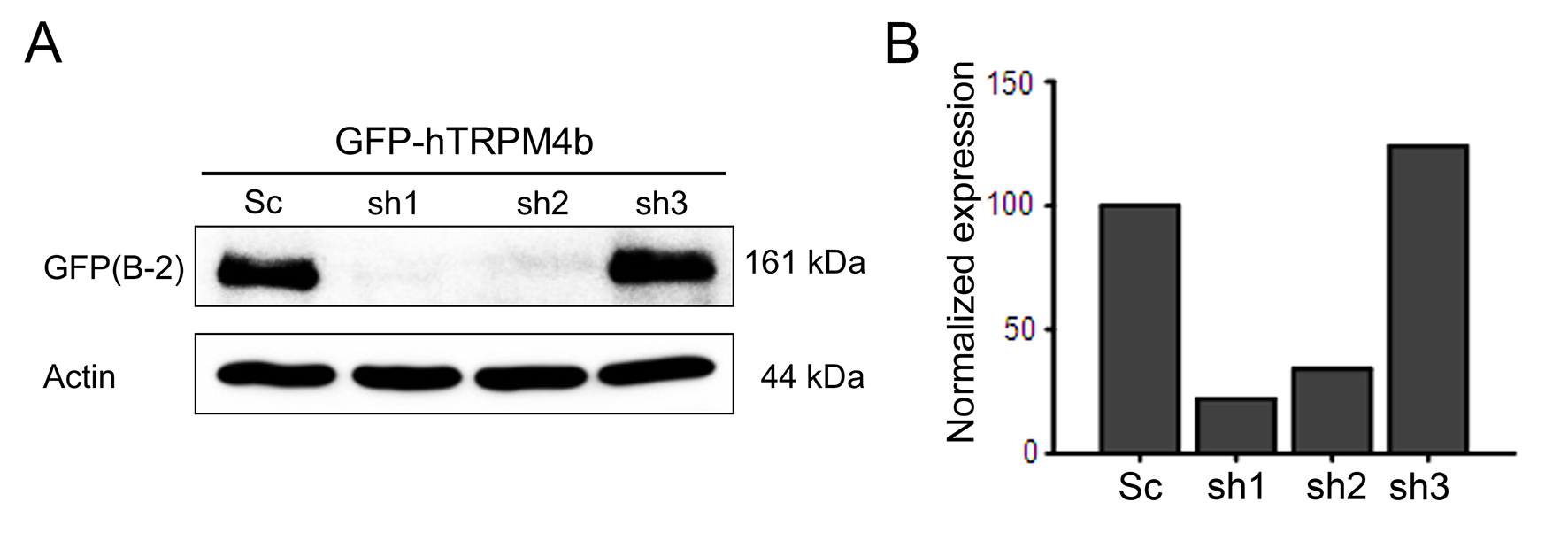

Supplement: Supplementary Figure 1 — Validation of TRPM4 shRNA constructs. (A) Typical WB results representing knockdown effects of the TRPM4 shRNA constructs in the HEK-293 overexpression system. (B) Quantification of results shown in (A). The results are representative of three independent experiments. [file Image_1.tif]

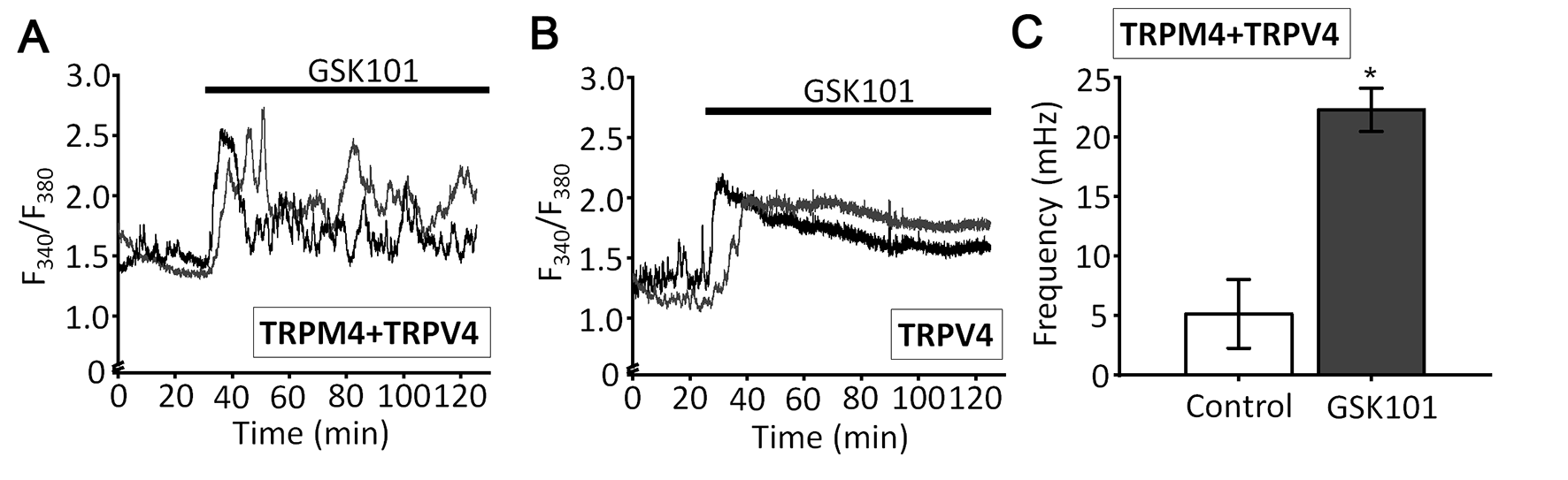

Supplement: Supplementary Figure 2 — HEK293 cells. TRPV4 and TRPM4 coexpression is sufficient to sustain calcium oscillations in the presence of GSK101. Representative traces of ratiometric signals in (A) TRPV4 + TRPM4 DNA-transfected cells and (B) Cells transfected with TRPV4 alone. (C) Averaged frequency of [Ca2+]i transients in TRPV4+TRPM4 expressing cells before and after GSK101 exposure. [file Image_2.tif]
